# Analysis of Prognostic Significance of CD47 Expression in Newly Diagnosed Large B Cell Lymphoma

**DOI:** 10.3390/life16050849

**Published:** 2026-05-20

**Authors:** Olivera Markovic, Ilija Bukurecki, Anica Divac Pravdic, Gligorije Marinkovic, Tatjana Terzic, Miroslav Pavlovic, Vladimir Nikolic, Nikola Vukosavljevic, Zorica Cvetkovic

**Affiliations:** 1 “Zvezdara” University Medical Center, 11000 Belgrade, Serbia; gligorije.marinkovic96@gmail.com (G.M.); pavlovic.miroslav982@gmail.com (M.P.); nvukosavljevic93@gmail.com (N.V.); 2 Faculty of Medicine, Belgrade University, 11000 Belgrade, Serbia; tatjana.terzic63@gmail.com (T.T.); vladimir.nikolic@med.bg.ac.rs (V.N.); zcvetkovic06@gmail.com (Z.C.); 3 “Bezanijska kosa” University Hospital Medical Center, Belgrade, 11000 Belgrade, Serbia; ilijabukurecki@gmail.com (I.B.); anicadivac92@gmail.com (A.D.P.); 4 Institute of Pathology, Faculty of Medicine, University of Belgrade, 11000 Belgrade, Serbia; 5 Institute of Epidemiology, Faculty of Medicine, University of Belgrade, 11000 Belgrade, Serbia; 6 “Zemun” Clinical Hospital Center, 11000 Belgrade, Serbia

**Keywords:** CD47+, LBCL, lymphoma

## Abstract

CD47 is a transmembrane protein that possesses the ability to inhibit macrophage phagocytosis, enabling immune system evasion. While CD47 overexpression is associated with a poor prognosis in solid malignancies, data on its prognostic significance in lymphomas are inconsistent. This study aimed to evaluate the clinical and prognostic significance of CD47 expression in patients with large B-cell lymphoma (LBCL). In this study biopsy specimens from 146 patients diagnosed with LBCL and treated with immunochemotherapy were analyzed. CD47 expression was assessed using standard immunohistochemical methods. A high level of CD47 expression was detected in 30 (20.5%) patients. High CD47 expression was more frequently observed in patients with high β2-microglobulin levels and extranodal disease compared to nodal LBCL. No significant difference in CD47 expression was observed between gastrointestinal LBCL and other extranodal localizations. CD47 positivity had no significant influence on progression-free survival (PFS) and overall survival (OS); however, a trend toward shorter PFS and OS was noted (*p* = 0.099 and *p* = 0.149, respectively). The median PFS and OS were 27 and 37 months, respectively, in patients with high CD47 expression, while the median PFS and OS were not reached in the group of CD47 negative patients. Although CD47 expression was not an independent predictor of survival, the magnitude and direction of the observed hazard ratios suggest a potentially clinically meaningful effect.

## 1. Introduction

Large B-cell lymphoma (LBCL) is the most common subtype of B-cell lymphoma, accounting for approximately 25–40% of all non-Hodgkin lymphomas [1]. A cure is achieved in about 50–60% of patients; however, a substantial proportion either exhibit primary resistance to immunochemotherapy or relapse after achieving remission [2,3,4,5].

Poor prognosis is associated with advanced disease, including large tumor burden, as well as biological characteristics that determine disease aggressiveness and resistance to therapy [1,2,3,4,5]. Although substantial progress has been made in understanding the biology of large B-cell lymphoma (LBCL), predicting the clinical course in individual patients remains challenging [6]. It is still difficult to identify, at diagnosis, those patients who are likely to have a poor prognosis. One of the key survival mechanisms of lymphoma cells and a major contributor to treatment failure is their ability to evade the immune response [7]. Specifically, reliable identification of patients at high risk of poor outcomes at the time of diagnosis is still limited. One of the key mechanisms underlying treatment failure is the ability of lymphoma cells to evade the host immune response [7]. Tumor immune escape occurs through multiple mechanisms, including impaired immune recognition, increased resistance to immune-mediated cell death, and the development of an immunosuppressive tumor microenvironment [7]. These processes are often driven by the expression of immunoregulatory molecules and cytokines that suppress antitumor immune responses and promote lymphoma cell survival. One such immunoregulatory molecule is CD47, which interacts with signal regulatory protein alpha (SIRPα) on macrophages and other immune cells, delivering a “do not eat me” signal that inhibits phagocytosis of malignant cells [7,8]. At the molecular level, CD47–SIRPα engagement leads to the activation of intracellular phosphatases, such as SHP-1 and SHP-2, which inhibit cytoskeletal rearrangements required for phagocytosis. This, in turn, reduces antigen presentation and weakens the activation of adaptive immune responses [9]. In addition, CD47 interacts with multiple ligands and intracellular signaling molecules, regulating downstream pathways involved in cell survival, proliferation, and immune modulation [9,10]. Over the past decade, CD47 overexpression has been documented in a broad spectrum of hematologic and solid malignancies [11,12,13,14,15]. Accumulating evidence indicates that elevated CD47 expression is associated with adverse prognostic features, including advanced disease stage, increased tumor burden, treatment resistance, and inferior overall survival across both hematologic malignancies, such as lymphoma and acute myeloid leukemia, and various solid tumors [11,12,13,14,15]. The aim of this study was to determine the prognostic significance of CD47 expression in patients with LBCL and to assess its association with clinical and histopathological parameters.

## 2. Materials and Methods

Biopsy specimens from 146 patients with LBCL diagnosed from January 2014 to September 2025 were obtained. The diagnosis was established according to the criteria of the World Health Organization classification [16]. The following clinical variables were analyzed: age, gender, clinical stage, ECOG, IPI, serum albumin, C-reactive protein, ß_2_-microglobulin, LDH, LBCL subtype (GCB/non-GCB), IPI, NCCN-IPI, lymphocyte and monocyte count, hemoglobin concentration and platelet count. The staging of the disease was done according to the Ann Arbor classification, the International Prognostic Index (IPI) and the NCCN-IPI scores which were determined, as described previously [3,17]. The patients were subdivided into GCB and non-GCB types according to the model proposed by Hans et al. [18].

### 2.1. Treatment

All patients had been treated with immunochemotherapy. The patients were treated with R-CHOP (rituximab, cyclophosphamide, doxorubicin, vincristine, prednisone) (130 patients, 89.04%), R-miniCHOP (rituximab, cyclophosphamide, doxorubicin, vincristine, prednisone) (7 patients, 4.79%), R-COEP (rituximab, cyclophosphamide, etoposide, prednisone) (3 patients, 2.05%), R-CVP (rituximab, cyclophosphamide, vincristine, prednisone) (4 patients, 2.73%) and R-EPOCH (rituximab, etoposide, prednisone, vincristine, cyclophosphamide, doxorubicin) (2 patients, 1.36%). Patients with clinical stages II–IV were treated with six to eight cycles of immunochemotherapy. Patients in the first clinical stage were treated with three cycles of immunochemotherapy and “involved”-field radiotherapy. Irradiation therapy (30–40 Gy) was applied after immunochemotherapy in patients with “bulky” disease or with residual disease. Treatment response was evaluated according to the Revised Response Criteria for Malignant Lymphoma [19].

### 2.2. Immunohistochemical Studies

Tumor tissue was obtained by biopsy of a lymph node or extranodal tissue, fixed in buffered formalin, pH 7.4 and embedded in paraffin. Four-micron-thick sections of formalin-fixed paraffin-embedded (FFPE) tissue blocks were cut and mounted on coated slides. Immunostaining was standardized using an appropriate external positive control of prostate tissue. A negative control was performed by omitting the primary antibody and following the standard staining procedure. The sections were deparaffinized in xylene, rehydrated in descending ethanol grades, and incubated for 5 min in 3% hydrogen peroxide to block endogenous tissue peroxidase. The immunoexpression of CD47 was assayed by means of the avidin/biotin/peroxidase complex method (Ultravision LP Detection system, Labvision) (Thermo Fisher Scientific, Kalamazoo, MI, USA) using DAB as a chromogen. The sections were stained with a CD47 antibody (clone CL6913, dilution 1:400, Sigma-Aldrich) (St. Louis, MO, USA). The antibody was incubated for half an hour at room temperature. After the development of the chromogen all slides were counterstained with hematoxylin.

### 2.3. Evaluation of IHC Samples

Slides were evaluated by two independent investigators (O.M., T.T.). in a blinded manner using an Olympus BX41 light microscope (Tokyo, Japan). Interobserver agreement was assessed, and in the case of disagreement, the observers reanalyzed the results until they reached a consensus. Cytoplasmic and/or membranous positivity was considered a positive result. The expression of the monoclonal antibody CD47 on tumor cells was analyzed semi-quantitatively as: score 0—no expression, score 1—discrete expression, score 2—weak expression, score 3—moderate expression, score 4—strong expression. Overall, expression levels with scores 0, 1, and 2 were considered low (CD47-low), while scores 3 and 4 were considered high (CD47-high), as previously described [20]. Interobserver agreement between the two pathologists for CD47 immunohistochemical scoring (0–4 scale) was assessed using Cohen’s kappa statistics. Given the ordinal nature of the data, both linear and quadratic weighted kappa coefficients were calculated, with quadratic weighting considered the primary measure. Agreement was interpreted according to Jacob Cohen’s criteria.

### 2.4. Statistical Analysis

All statistical analyses were performed using SPSS version 26.0 (SPSS Inc., Chicago, IL, USA), with a significance level set at *p* < 0.05. Categorical variables were presented as frequencies and continuous variables as the median along with the minimal and maximal values. The normality of distribution was assessed using the Kolmogorov–Smirnov test. The association between CD47 expression and categorical variables was assessed using the chi-square test. Survival outcomes were analyzed using the Kaplan–Meier method, and differences between groups were assessed using the log-rank test. Hazard ratios were estimated using Cox proportional hazards regression models. Survival data were analyzed using the Kaplan–Meier method with right-censoring, where patients without an event at the end of follow-up were considered censored. The median progression-free survival (PFS) and overall survival (OS) were estimated for the patients with high CD47 and low CD47 expression. The mean survival time with 95% confidence intervals (CI) was also calculated. To assess potential predictors of CD47 expression and its association with clinical and laboratory parameters, univariate and multivariate Cox proportional hazards regression model were conducted. Hazard ratios (HRs) with 95% CIs were reported for significant predictors. Variables included in the multivariable Cox proportional hazards regression model were selected based on clinical relevance, biological plausibility, and statistical significance in the univariate analysis. To assess potential multicollinearity among covariates, the variance inflation factor (VIF) and tolerance statistics were evaluated before model construction. No significant multicollinearity was detected among the variables included in the final multivariable model. Survival data were analyzed as right-censored, where patients who did not experience the event of interest during the follow-up period were treated as censored at the time of last contact.

## 3. Results

### 3.1. Patient’s Characteristics

Clinical data for all patients are summarized in Table 1. Among the analyzed cases, most patients (*n* = 139) were diagnosed with LBCL, NOS. Less frequent subtypes included primary cutaneous LBCL, leg type (*n* = 3), T-cell/histiocyte-rich LBCL (*n* = 1), EBV-positive LBCL (*n* = 1), and primary mediastinal large B-cell lymphoma (*n* = 2).

### 3.2. Immunohistochemical Analysis

**Figure 1:**
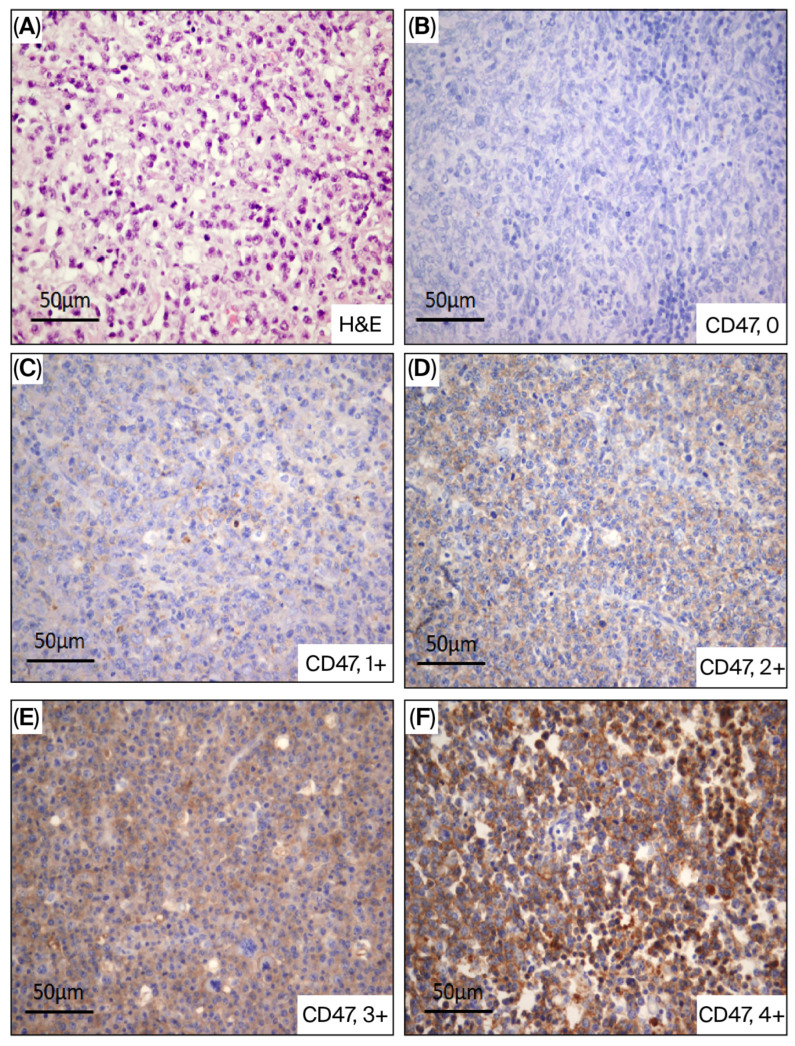
CD47 expression in biopsy specimens: (**A**) H&E staining of tumor tissue, (**B**) The LBCL cells are negative (score 0), (**C**) Discrete positivity (score I) (CD47 low), (**D**) Weak positivity (score II), (**E**) Moderate cytoplasmic and/or membranous positivity (score III), (**F**) Strong cytoplasmic and/or membranous positivity (score IV) (Score III and IV—CD47 high) (streptavidin-biotin, ×400).

High CD47 expression (scores 3 and 4) was detected in 30 (20.5%) patients while low CD47 (scores 0, 1 and 2) was found in 116 (79.5%) patients. Staining was manifested in the form of cytoplasmic and/or membranous positivity (Figure 1). Quadratic weighted Cohen’s kappa demonstrated almost perfect agreement between observers (κ = 0.87, 95% CI 0.82–0.91, *p* < 0.001), while linear weighted kappa showed slightly lower but still substantial agreement (κ = 0.79, 95% CI 0.73–0.85, *p* < 0.001), reflecting minor discrepancies predominantly between adjacent scoring categories. Most discrepancies were limited to their adjacent categories, particularly between scores 1 and 2 and between scores 3 and 4, with no major discordances observed.

### 3.3. Correlation Between CD47 Immunoexpression and Subtype and Clinical Parameters

High CD47 expression was more frequently observed in patients with high β2-microglobulin levels (*p* = 0.02) and extranodal disease compared to nodal LBCL (*p* = 0.01). No significant difference in CD47 expression was observed between gastrointestinal LBCL (25%) and LBCL arising in other extranodal locations (33%). CD47 expression did not correlate significantly with age, sex, ECOG performance status, CRP levels, lymphocyte and monocyte counts, IPI, NCCN-IPI, “bulky” disease, hemoglobin levels, LDH and GCB/non-GCB subtype (Table 2).

Therapy response was achieved in 75.3% of patients. There was no difference in therapy response to immunochemotherapy according to CD47 expression. However, in the group of patients with high CD47 expression within the non-GCB subtype of LBCL, therapy response was achieved in 16 (69.6%) patients, while in the group of GCB LBCL, therapy response was achieved in 5 (83.3%) patients (*p* = 0.502).

### 3.4. Prognostic Significance of CD47 Immunoexpression for OS

**Figure 2:**
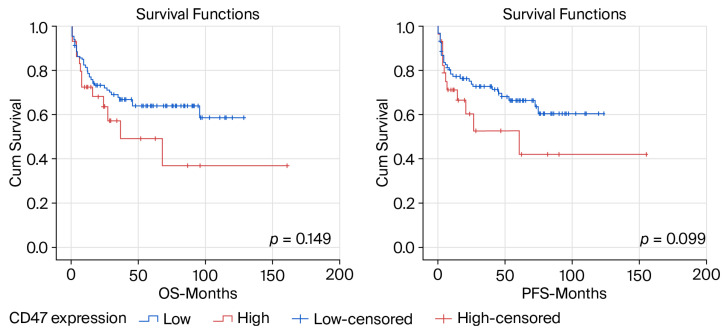
Kaplan–Meier OS and PFS curves according to CD47 expression.

The median follow-up period for OS was 46 months (range 1 to 121 months). At the time of the final analysis, 94 (64.5%) patients were alive. In the group of patients with low CD47 expression, 77 (66.4%) of them were alive, while in the group of those with high CD47 expression 17 (56.7%) patients were alive (*p* = 0.322). The median survival of the entire group of analyzed patients was not reached. The median survival of patients with high CD47 expression was 37.0 months, while the median survival of those with low CD47 expression was not reached (*p* = 0.149, 95% CI 23.53 (0.00–82.771)) (Figure 2). The median PFS of the patients with high CD47 expression was 27 months, while the median PFS of those with low CD47 expression was not reached (*p* = 0.099, 23.53 (0.00–73.122)). Kaplan–Meier PFS and OS curves are shown in Figure 2.

**Figure 3:**
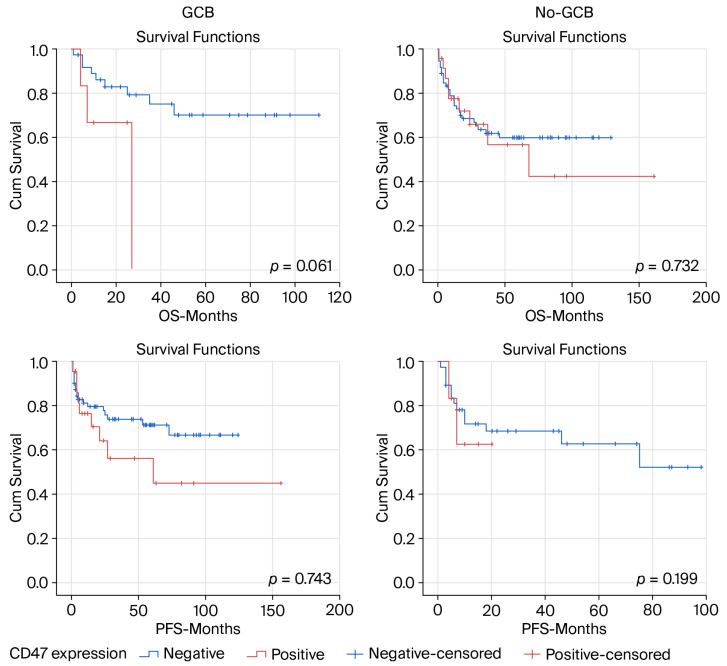
Kaplan–Meier curves for overall and progression-free survival according to CD47 expression stratified by COO subtype (GCB and non-GCB).

In the GCB subgroup, patients with high CD47 expression tended to have shorter overall survival compared with those with low CD47 expression, with a mean OS of 19.8 months (95% CI 9.9–29.8) versus 83.7 months (95% CI 68.5–99.0), although the difference did not reach statistical significance (log-rank *p* = 0.061) (Figure 3). A similar pattern was observed for progression-free survival, where CD47-high patients had a mean PFS of 14.6 months (95% CI 8.7–20.6) compared with 63.8 months (95% CI 49.2–78.4) in CD47-low patients; however, this difference was also not statistically significant (log-rank *p* = 0.743). In the non-GCB subgroup, no significant association between CD47 expression and survival outcomes was observed. Mean overall survival was 84.9 months (95% CI 48.2–121.5) in CD47-high patients and 82.3 months (95% CI 68.5–96.1) in CD47-low patients (log-rank *p* = 0.732). Similarly, mean progression-free survival was 82.2 months (95% CI 47.2–117.1) in the CD47-high group and 89.6 months (95% CI 76.5–102.7) in the CD47-low group, without statistically significant differences (log-rank *p* = 0.199) (Figure 3). Findings from subgroup analyses should be interpreted cautiously due to the limited number of CD47-high cases.

In multivariable Cox regression analysis, both NCCN-IPI (HR 1.56, 95% CI 1.10–2.23, *p* = 0.013) and bulky disease (HR 2.38, 95% CI 1.05–5.40, *p* = 0.039) were independently associated with shorter progression-free survival. CD47 expression was not independently associated with PFS, although the direction of the effect suggested a potential adverse impact (Table 3).

In multivariate analysis, bulky disease remained an independent predictor of worse overall survival (HR 6.35, 95% CI 1.48–27.17, *p* = 0.013), as did the NCCN-IPI score (HR 2.29, 95% CI 1.11–4.75, *p* = 0.025). Lower albumin levels also remained independently associated with worse survival (HR 0.87, 95% CI 0.79–0.96, *p* = 0.006). β2-microglobulin showed a borderline association in the multivariate model (HR 0.69, 95% CI 0.48–1.01, *p* = 0.054). CD47 expression was not significantly associated with overall survival in this analysis (Table 4).

## 4. Discussion

CD47 is a ubiquitously expressed cell surface protein that inhibits phagocytosis by macrophages. Its interaction with signal-regulatory protein alpha (SIRPα) on macrophages delivers a “do not eat me” signal, enabling cancer cells to evade macrophage-mediated clearance [21]. In addition to this role, CD47 exerts multiple functions independent of SIRPα-mediated phagocytosis. It interacts with other molecules like thrombospondin, various integrins, VEGFR2 and NOX1 as well as a number of cytoplasmic signaling molecules regulating downstream signaling pathways involved in the response to oxidative stress, cell death, metabolism, adhesion, motility, migration, proliferation and the regulation of immune responses [9,10]. The functional outcome of CD47 signaling depends on the interacting partner, cell type, and microenvironment [9]. CD47/TSP-1 interaction promotes the survival of T cell lymphoma cells in vitro and enhances tumor growth in vivo [22]. The interaction between CD47 and other molecular partners contributes to T-cell exhaustion and the suppression of T-cell effector functions [23].

CD47 is overexpressed in a wide range of hematological and solid malignancies. The highest proportion and intensity of CD47-positive lymphoma cells have been reported in small B-cell lymphomas, including mantle cell lymphoma, marginal zone lymphoma, and follicular lymphoma. In contrast, moderate CD47 expression has been observed in T- and B-lymphoblastic lymphoma, Hodgkin lymphoma, diffuse large B-cell lymphoma (DLBCL), peripheral T-cell lymphoma, γδ T-cell lymphoma, and angioimmunoblastic T-cell lymphoma [22,24,25,26].

The reported frequency of CD47 expression in patients with large B-cell lymphoma (LBCL) varies widely across studies, ranging from 8.3% to 48%, likely reflecting differences in anatomical locations and disease subtypes [20,27,28,29,30]. Notably, a higher frequency of strong CD47 expression has been described in intestinal LBCL compared with other anatomical sites, suggesting a potential role of CD47 in extranodal dissemination [27]. In our study, a higher frequency of elevated CD47 expression was observed in extranodal lymphomas compared with nodal LBCL. However, no significant differences in CD47 expression were found between intestinal LBCL and other extranodal sites.

Previous studies have demonstrated considerable variability in CD47 expression in large B-cell lymphoma (LBCL) [20,27,29,30]. Shen et al. reported CD47 positivity in 84.9% of cases; however, strong and diffuse expression (IHC score ≥ 2) was observed in only 11% of patients [29]. Similarly, Lee et al. identified high CD47 expression in 11% of newly diagnosed LBCL cases [20]. In contrast, Marra et al. reported a substantially higher frequency of CD47 overexpression (48%), with significantly greater expression in the non-GCB compared with the GCB subtype [30]. This association between elevated CD47 expression and the non-GCB subtype has also been confirmed in other studies [20,27]. In line with these findings, our study demonstrated a higher frequency of CD47 expression in the non-GCB group; however, this difference did not reach statistical significance, likely due to the limited sample size. The wide variability in reported CD47 expression across studies may reflect underlying biological heterogeneity, as well as methodological differences, including antibody selection, scoring systems, and cut-off definitions. Importantly, the high interobserver agreement observed in our study indicates that CD47 immunohistochemical assessment using a 0–4 scoring system is reproducible and reliable, supporting its potential utility in both research settings and clinical stratification without significant observer-dependent bias.

Bouwstra et al. demonstrated that high CD47 expression may compromise the efficacy of immunochemotherapy in patients with non-GCB LBCL, suggesting a potential role in treatment resistance and poorer clinical outcomes [31]. In our cohort, patients with high CD47 expression within the non-GCB subgroup showed a trend toward lower response rates. However, the small number of cases with elevated CD47 expression limits the strength of this observation and precludes definitive conclusions.

Elevated CD47 expression has also been identified as a prognostic marker in solid malignancies. It has been shown to represent an independent predictor of recurrence in resected non–small cell lung cancer and to be associated with poor prognosis in clear cell renal cell carcinoma following curative resection [8,11]. Furthermore, a recent meta-analysis demonstrated that high CD47 expression correlates with adverse clinical outcomes, including advanced disease stage, poor tumor differentiation, and an increased risk of recurrence, particularly in malignancies of the digestive and respiratory systems [15].

Recent studies have further highlighted the prognostic relevance of CD47 across hematologic malignancies. Sammartano et al. (2025) reported shorter survival in patients with acute myeloid leukemia exhibiting CD47 expression detected by flow cytometry [13]. In follicular lymphoma, Kume et al. demonstrated that high CD47 expression was associated with inferior progression-free survival (PFS) at 12 and 24 months, although no significant impact on overall survival (OS) was observed [25]. Similarly, CD47 expression has been identified as a significant predictor of both shorter PFS and OS in Hodgkin lymphoma [26].

In large B-cell lymphoma (LBCL), Shen et al. (2024) reported that elevated CD47 expression was associated with increased infiltration of tumor-associated M2 macrophages, shorter PFS and OS, and enhanced activity of oncogenic and immune-regulatory pathways, including cytokine signaling [29]. They also observed a higher prevalence of CD47 expression in MCD-like and N1-like molecular subtypes of LBCL [29].

However, the prognostic significance of CD47 expression in LBCL remains inconsistent. Some studies suggest an adverse prognostic impact limited to specific subgroups, such as patients with the non-GCB subtype [31] or those with non-intestinal extranodal disease [27], while others, including Lee et al., found no significant association between CD47 expression and outcomes in patients treated with R-CHOP [20]. In our cohort, although not reaching statistical significance, high CD47 expression was associated with a trend toward shorter PFS and OS. These findings should be interpreted with caution, given the limited sample size, but may further support a context-dependent role of CD47 in lymphoma biology and prognosis. Notably, given that most patients in the cohort had LBCL, NOS, the impact of other subtypes on the overall findings was likely minimal. These cases were managed using the same standard-of-care treatment protocols applied to the remainder of the cohort, with no systematic differences in therapeutic approaches that would be expected to introduce meaningful bias.

Exploratory analyses stratified by COO subtype did not demonstrate statistically significant differences in progression-free survival according to CD47 expression. A similar numerical pattern toward shorter PFS in CD47-high patients was observed in both subgroups. In the GCB cohort, a visual separation of survival curves was noted; however, this finding is based on a very small number of patients and events and is therefore highly unstable and likely subject to random variation. In the non-GCB subgroup, survival curves were largely overlapping. Overall, these subgroup analyses are underpowered and do not support any definitive conclusions regarding the prognostic impact of CD47 within COO-defined subgroups.

The variability in reported prognostic significance of CD47 expression is likely multifactorial. It may reflect context-dependent biological effects of CD47 within distinct tumor microenvironments, as well as the influence of co-occurring genetic alterations that modulate pathways functionally linked to CD47 signaling. In addition, heterogeneity in immunohistochemical methodologies, including antibody selection, scoring systems, and cut-off definitions, may further contribute to inconsistent findings across studies. Importantly, immune evasion mechanisms independent of CD47 may also play a more dominant role in suppressing antitumor immunity in certain settings. In this context, Chung et al. demonstrated that sialylated CD43 exerts an even stronger inhibitory effect on macrophage function than CD47 and represents a key regulator of antileukemic immunity in human acute myeloid leukemia [32].

Blocking the CD47–SIRPα interaction and thereby restoring phagocytosis represents a promising therapeutic strategy that harnesses the innate immune system to enhance the elimination of malignant cells [33]. Several CD47-targeting agents have been developed and are currently being evaluated in various phases of clinical trials. However, given the ubiquitous expression of CD47 on normal cells, early anti-CD47 monoclonal antibodies were associated with significant on-target toxicities [34]. To overcome these limitations, novel therapeutic approaches with improved specificity and reduced toxicity have been developed, including bispecific antibodies, SIRPα–Fc fusion proteins, and small-molecule inhibitors. Emerging clinical data suggest that CD47-targeted therapy, either as monotherapy or in combination regimens, may induce durable complete responses in patients with relapsed or refractory LBCL [35,36,37,38]. Nevertheless, comprehensive characterization of CD47 expression in relation to clinicopathological features is required to better define patient subsets most likely to benefit from this therapeutic approach.

This study contributes to clarifying the prognostic significance of CD47 expression in patients with LBCL, an area characterized by inconsistent findings in the current literature. However, several limitations should be acknowledged. First, the retrospective design introduces a potential risk of selection bias. Second, heterogeneity in treatment regimens may have influenced response rates and survival outcomes. Third, the relatively small sample size, particularly within subgroup analyses, limits statistical power and the robustness of the conclusions. Larger, prospective studies are warranted to further define the prognostic and predictive value of CD47 in LBCL and to help identify patients who may benefit from CD47-targeted therapeutic strategies.

## 5. Conclusions

Although CD47 expression was not identified as an independent predictor of survival in the present study, the lack of statistical significance may be related, at least in part, to the limited number of CD47-high patients included in the analysis. Therefore, additional studies with larger sample sizes are needed to better clarify the prognostic significance of CD47 expression in LBCL patients and its potential role in identifying candidates for anti-CD47 antibody therapy.

## Figures and Tables

**Table 1:** Clinical Data and Histological Features of 146 de novo LBCL patients.

Category	Variable	Value
Demographic characteristics		
	Age, median (range)	67 (22–91)
	Age > 60 years	102 (69.9%)
	Gender (male/female)	85 (58.2%)/61 (41.8%)
Disease stage		
	I	13 (8.9%)
	II	49 (33.6%)
	III	33 (22.6%)
	IV	51 (34.9%)
ECOG performance status		
	0	45 (30.8%)
	1	63 (43.2%)
	2	25 (17.1%)
	3	7 (4.8%)
	4	6 (4.1%)
Clinical prognostic factors		
	B symptoms	69 (47.3%)
	Extranodal involvement	49 (33.6%)
	Bulky disease ≥ 7 cm	72 (49.3%)
	Bulky disease ≥ 10 cm	29 (19.9%)
IPI score		
	Low	40 (27.4%)
	Low-intermediate	41 (28.1%)
	High-intermediate	36 (24.7%)
	High	29 (19.9%)
NCCN-IPI score		
	Low	17 (11.7%)
	Low-intermediate	35 (24.1%)
	High-intermediate	55 (37.9%)
	High	38 (26.2%)
Laboratory parameters		
	LDH (U/L)	447.0 (504), 210–6151
	β2-microglobulin (mg/L)	3.1 (2), 1–20
	CRP (mg/L)	21.9 (48), 1–214
	Lymphocytes (×10^9^/L)	1.3 (1), 0.1–16
	Monocytes (×10^9^/L)	0.5 (0.3), 0.04–3.6
Biological subtype		
	GCB	43 (29.5%)
	Non-GCB	94 (64.4%)
	Unclassified	9 (6.2%)
Treatment response		
	CR	103 (70.6%)
	PR	8 (5.5%)
	MR	3 (2.1%)
	SD	4 (2.7%)
	PD	17 (11.6%)

**Table 2:** The expression of CD47 according to demographic and clinical characteristics of the patients.

Variable	Total *n* (%)	CD47 Low *n* (%)	CD47 High *n* (%)	*p* Value
Sex				
Female	61 (41.8)	47 (77.0)	14 (23.0)	0.543
Male	85 (58.2)	69 (81.2)	16 (18.8)	
Age, median (range)	67 (22–91)	67 (22–91)	69 (40–85)	0.615
≤60 years	44 (30.1)	36 (81.8)	8 (18.2)	0.642
>60 years	102 (69.9)	80 (78.4)	22 (21.6)	
ECOG				
0	45 (30.8)	37 (82.2)	8 (17.8)	0.957
1	63 (43.2)	49 (77.8)	14 (22.2)	
2	25 (17.1)	20 (80.0)	5 (20.0)	
3	7 (4.8)	5 (71.4)	2 (28.6)	
4	6 (4.1)	5 (83.3)	1 (16.7)	
CRP				
≤5.1	46 (33.3)	37 (80.4)	9 (19.6)	0.768
>5.1	92 (66.7)	72 (78.3)	20 (21.7)	
β2-microglobulin				
≤2.2 mg/L	39 (33.3)	35 (89.7)	4 (10.3)	0.020
>2.2 mg/L	78 (66.7)	55 (70.5)	23 (29.5)	
Lymphocytes (×10^9^/L)				
≤1	48 (33.1)	37 (77.1)	11 (22.9)	0.641
>1	97 (66.9)	78 (80.4)	19 (19.6)	
Monocytes (×10^9^/L)				
≤0.5	69 (48.6)	59 (85.5)	10 (14.5)	0.060
>0.5	73 (51.4)	53 (72.6)	20 (27.4)	
Bulky disease				
<7 cm	53 (66.3)	43 (81.1)	10 (18.9)	0.076
≥7 cm	27 (33.8)	17 (63.0)	10 (37.0)	
IPI				
Low	40 (28.4)	34 (85.0)	6 (15.0)	0.712
Low-intermediate	39 (27.7)	30 (76.9)	9 (23.1)	
High-intermediate	33 (23.4)	25 (75.8)	8 (24.2)	
High	29 (20.6)	24 (82.8)	5 (17.2)	
RIPI				
Very good	20 (13.8)	15 (75.0)	5 (25.0)	0.538
Good	63 (43.4)	53 (84.1)	10 (15.9)	
Poor	62 (42.8)	48 (77.4)	14 (22.6)	
NCCN-IPI				
Low	17 (11.7)	15 (88.2)	2 (11.8)	0.710
Low-intermediate	35 (24.1)	29 (82.9)	6 (17.1)	
High-intermediate	55 (37.9)	42 (76.4)	13 (23.6)	
High	38 (26.2)	30 (78.9)	8 (21.1)	
Extranodal involvement	49 (33.6)	33 (67.3)	16 (32.7)	0.010
LDH (≥428 U/L)	77 (52.7)	57 (74.0)	20 (26.0)	0.087
Subtype				
GCB	43 (31.4)	37 (86.0)	6 (14.0)	0.162
Non-GCB	94 (68.6)	71 (75.5)	23 (24.5)	
Therapy response				
Yes	110 (75.3)	89 (80.9)	21 (19.1)	0.446
No	36 (24.7)	27 (75.0)	9 (25.0)	

**Table 3:** Univariate and multivariate analysis of PFS in the studied group of patients.

Clinical Characteristics	Univariate Analysis		Multivariate Analysis	
	*p*	HR (CI 95%)	*p*	HR (CI 95%)
Albumin	<0.001	0.91 (0.87–0.96)	0.007	0.88 (0.81–0.97)
β2-microglobulin	0.005	1.11 (1.00–1.20)	0.020	1.29 (1.04–1.61)
“Bulky” disease (>7 cm)	0.003	2.98 (1.47–6.07)		
NCCN-IPI	<0.001	1.86 (1.36–2.56)		
CD47 high expression	0.106	1.66 (0.90–3.05)		
Hgb	0.063	0.99 (0.97–1.00)		
Lymphocytes	0.016	0.54 (0.32–0.89)		
Monocytes	0.807	0.87 (0.29–2.61)		
Thrombocytes	0.853	1.00 (1.00–1.00)		
CRP	0.699	1.00 (1.00–1.00)		

**Table 4:** Univariate and multivariate analysis of OS in the studied group of patients.

Clinical Characteristics	Univariate Analysis		Multivariate Analysis	
	*p*	HR (95% CI)	*p*	HR (95% CI)
Bulky disease (>7 cm)	0.012	2.59 (1.23–5.45)	0.013	6.35 (1.48–27.17)
Albumin	<0.001	0.87 (0.83–0.91)	0.006	0.87 (0.79–0.96)
β2-microglobulin	0.001	1.12 (1.04–1.20)	0.054	0.69 (0.48–1.01)
Hgb	0.002	0.98 (0.97–0.99)		
Lymphocytes	0.006	0.50 (0.31–0.82)		
Monocytes	0.417	0.68 (0.27–1.71)		
Thrombocytes	0.323	1.00 (1.00–1.00)		
CRP	0.682	1.00 (1.00–1.00)		

## Data Availability

The data presented in this study are available on request from the corresponding author due to patient privacy.

## Abbreviations

The following abbreviations are used in this manuscript:

CD	Cluster of differentiation
LBCL	Large B-cell lymphoma
NHL	Non-Hodgkin lymphoma
SIRPα	Signal regulatory protein alpha
ECOG	Eastern cooperative oncology group
LDH	Lactate dehydrogenase
H&E	Hematoxylin and Eosin
IPI	International prognostic index
R-IPI	Revised International prognostic index
NCCN-IPI	The National Comprehensive Cancer Network International prognostic index
GCB	Germinal center B-cell like
FFPE	Formalin-fixed paraffin embedded
DAB	Diaminobenzidine
PFS	Progression-free survival
OS	Overall survival
CI	Confidence interval
OR	Odds ratio
CRP	C-reactive protein
VEGFR-2	Vascular Endothelial Growth Factor Receptor 2
NOX-1	reduced nicotinamide adenine dinucleotide phosphate oxidase 1
TSP-1	Thrombospondin-1
EN	Extra-nodal
COO	Cell of Origin
IHC	Immunohistochemistry
AML	Acute myeloid leukemia
